# High-intensity power-resolved radiation imaging of an operational nuclear reactor

**DOI:** 10.1038/ncomms9592

**Published:** 2015-10-09

**Authors:** Jonathan S. Beaumont, Matthew P. Mellor, Mario Villa, Malcolm J. Joyce

**Affiliations:** 1 Department of Engineering, Gillow Avenue, Lancaster University, Lancaster, LA1 4YW, UK; 2 Createc Ltd., Derwent Mills Commercial Park, Cockermouth CA13 0HT, UK; 3 Atominstitut, Vienna University of Technology, 1020 Vienna, Austria; 4 Hybrid Instruments Ltd., Unit 16, ICT Centre, Birmingham Research Park, Vincent Drive, Edgbaston B15 2SQ, UK

## Abstract

Knowledge of the neutron distribution in a nuclear reactor is necessary to ensure the safe and efficient burnup of reactor fuel. Currently these measurements are performed by in-core systems in what are extremely hostile environments and in most reactor accident scenarios it is likely that these systems would be damaged. Here we present a compact and portable radiation imaging system with the ability to image high-intensity fast-neutron and gamma-ray fields simultaneously. This system has been deployed to image radiation fields emitted during the operation of a TRIGA test reactor allowing a spatial visualization of the internal reactor conditions to be obtained. The imaged flux in each case is found to scale linearly with reactor power indicating that this method may be used for power-resolved reactor monitoring and for the assay of ongoing nuclear criticalities in damaged nuclear reactors.

## Abstract (web summary)

Monitoring the activity of nuclear reactors requires measuring the neutron distribution in the core efficiently and in real time. Here, the authors present an imaging approach for neutrons and gamma-rays that thanks to a slit-pupil-like design, enables radiations to be visualized directly in operative reactors.

## Graphical abstract

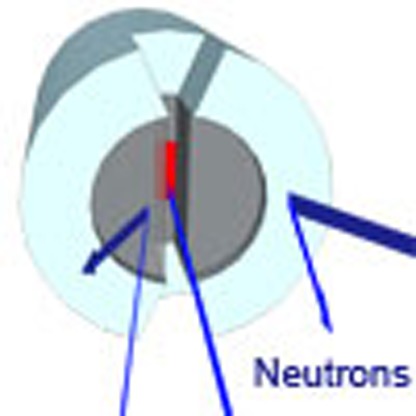

Nuclear reactors are controlled by shaping the population of neutrons within the core^1^. This distribution needs to be measured regularly and accurately to infer power and to ensure the safe and efficient burnup of reactor fuel. The fission process produces highly penetrating neutrons and gamma rays inside the reactor, some of which can be detected far from their point of origin. These radiations therefore can be imaged to yield information of the internal reactor conditions, independently from instrumentation within the core. Several levels of redundancy are needed for reactor monitoring due to the harsh environment associated with the fission process. Monitoring systems, such as fission chambers and rhodium detector systems are expensive and have limited lifespans. They face severe challenges associated with the in-core environment to remain viable, including high temperatures, corrosion, extreme radiation levels and miniaturization. In-core monitoring systems often have short lifespans relative to the expected life of modern reactors, are expensive, difficult to replace and can be destroyed easily in the event of a core-damage accident^2^.

In the context of major reactor accidents, the most significant of late is that of the Fukushima Daiichi disaster. Much research has been reported, and a variety of imaging methods have been used to image contamination from this incident in the natural environment^3^. These reports frequently exploit the use of Compton cameras that only respond to gamma radiation and are not resilient to high-intensity fields. Thus they are not easily transferable to power-resolved imaging or operational reactors that require assay of the neutron emission, let alone the immediate aftermath post-accident. There have been complementary reports of systems for internal inspection of failed reactor plant^4^ but these are intrusive and provide for the survey of fuel debris inside reactor plant once access to a stricken reactor is achieved. In terms of imaging the stricken reactor itself, the most evolved technique is the use of cosmic-ray muons^5^ that can non-intrusively reveal the distribution of fuel debris in a reactor, though not the reactivity profile. However, the major disadvantage of this approach is the extensive exposure time of the order of months. This precludes the assay of reactor systems in operation or power-resolved images as presented here, which require timescales of the order of minutes to hours. Most importantly, analogous to the comparison of X-ray transmission imaging with single-photon emission computerized tomography in the medical field, our report highlights the benefit of *in situ* radiation imaging over long-exposure, transmission imaging. The former yields reactivity information in addition to fuel layout whereas the latter only yields information on the distribution of material in the core. Subtle asymmetries in the power distribution can lead to economic under-performance in commercial power reactors, and can influence experimental facilities that rely on small-scale test reactors for sample irradiation or neutron radiography^6^,^7^. Accounting for the axial offset anomaly experienced with the operation of pressurized water reactors^8^, in which the neutron distribution differs from predicted estimates, could be aided by high-intensity imaging technology.

We present an approach to reactor monitoring using a stand-off imaging system, able to image fast-neutron and gamma-ray fields emitted by a reactor core. This method is passive, non-intrusive and does not require penetration into the core containment. The system is lightweight and portable, weighing a total of 20 kg, and will fit inside a small suitcase. The simplicity and compactness of this single-detector system^9^ overcomes the problems of other imagers reported in the literature that are not portable and use large arrays of detectors^10^,^11^, and therefore are not easily transferable to situations described here. Imaging high-intensity radiation fields currently has challenges, some of which have been addressed in nature. Slit pupils in the eyes of some terrestrial vertebrates, including some cats, are known to be oriented orthogonal to the visual streak^12^. This adaptation extends the useful visual range into high-intensity light levels^13^. In analogy this approach has been applied here by using a slit collimator that creates a thin band of spatial sensitivity, which prevents detector saturation and additionally preserves angular resolution. Slit collimators of similar designs have been used to image gamma-ray fields^14^,^15^ though have not been applied to more highly penetrating fast neutrons. This system provides a method of spatially dependent monitoring that is uniquely not in-core. Here we show the first images of a functioning nuclear reactor produced from radiation emitted directly from the core as a result of the fission process. In these images the isolation of the neutron field almost exclusively discriminates information about nuclear fuel material. Further, the results indicate that this imaging approach is power-resolved, such that the images reveal information of the reaction rates, that is, the rate of fuel burnup within the core. This capability may lead to a new widely used method of reactor monitoring with relevance spanning small medical isotope reactors through to the large power reactors currently in build worldwide, many of which emit a sufficient fast-neutron component^16^. This approach may be of merit to the new generation of small modular reactor designs^17^ where longer lifecycles might require lifetimes beyond that of today's monitoring systems. We foresee the provision of continuous insight throughout the lifetime of a reactor, post-defueling to include decommissioning. This approach could also inform our view of internal conditions in the case of a nuclear accident where other monitoring systems will often have been destroyed. Further applications of this mixed-field imaging system are relevant to any other neutron-emitting systems and materials, including nuclear fusion research, and could herald the way for stand-off, non-intrusive enrichment assessment.

## Results

### Deployment and calibration

**Figure 1:**
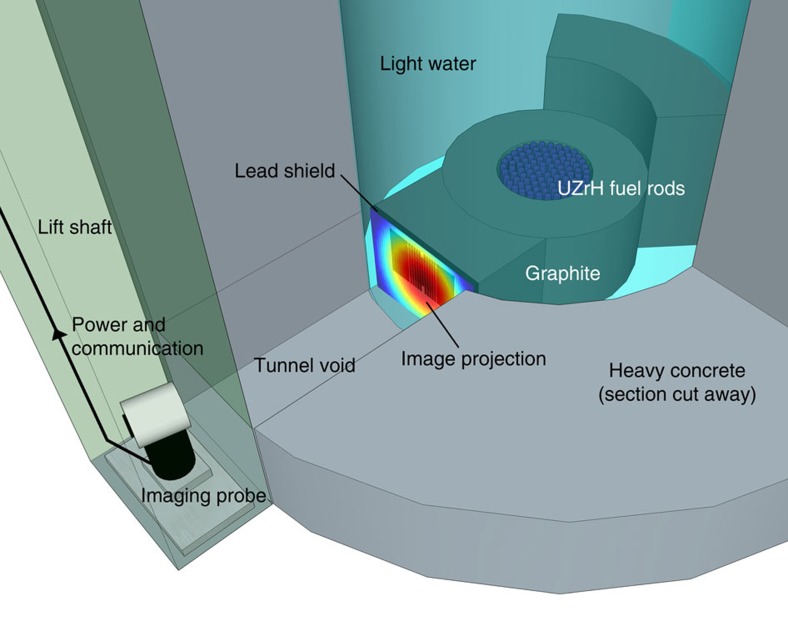
Geometry of reactor. A schematic of the imaging probe as described for the experimental set-up and geometry of the reactor core and materials. Access to the core was provided by a lift shaft usually used for transporting irradiation samples. Power and communications cabling linked the probe with data acquisition electronics and associated equipment. The detector position, that is, the image origin was located 25 cm vertically and 229 cm horizontally from the geometric centre of the core. A cuboidal air void labelled ‘tunnel void' located between the probe and the water coolant allowed the unshielded passage of radiation from the core region to the probe. A projection of a neutron image obtained in this work is included to provide a visualization of the information the technique provides.

**Figure 2:**
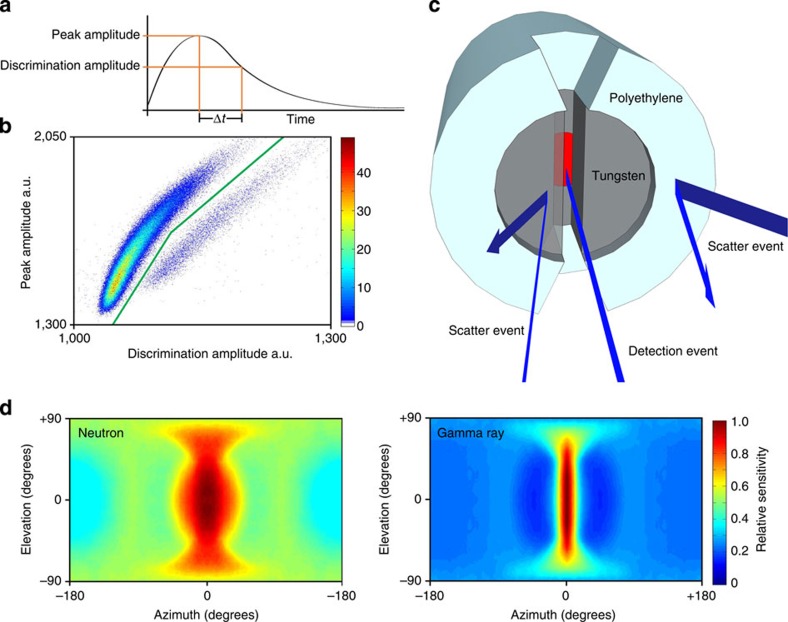
Imaging parameters and slit shielding. (**a**) An illustration of the discrimination algorithm operating on a detector pulse after the application of a moving average algorithm. This algorithm was used to produce plot **b**. (**b**) Discrimination plot showing radiation events from reactor imaging after the application of the discrimination algorithm. Neutron and gamma-ray events are seen to cluster in plumes. The discrimination line used to separate neutron and gamma-ray events is shown on the plot. Discrimination was performed in real time. (**c**) Schematic illustration of the collimator and detector (red) with examples of radiation interactions. The collimator has been designed to provide a spatial sensitivity bias to neutrons and gamma rays such that radiations incident on the slit are more likely to be detected than those incident on shielded regions. (**d**) Neutron and gamma-ray system matrix elements which map the sensitivity of the collimated detector, as shown in **c** to all space. The sensitivity region in each case roughly represents a thin band extending from −90° to+90° in elevation.

The irradiation room of the reactor was accessed by the use of a narrow (∼30 × 30 cm) lift shaft, which was used to lower the probe into position in proximity of the core (Fig. 1). Once in place the reactor was brought to a power of 10 kW and data were accumulated in the mixed-field analyser graphical user interface for 10 min. The system uses a single detector, containing 6 ml of the liquid scintillant EJ-301 (ref. 18) which fluoresces under exposure to fast neutrons and gamma rays and does not suffer neutron damage allowing extended use. During data collection a mixed-field analyser unit performs a discrimination algorithm on the detected radiation events (Fig. 2a,b), outputting real-time pulses that identify neutrons and gamma rays^19^,^20^. The plumes of discriminated neutrons and gamma rays were observed to have good separation and the discrimination parameters were set. These parameters were tested before and after each image and were not observed to change significantly.

### Reactor imaging

**Figure 3:**
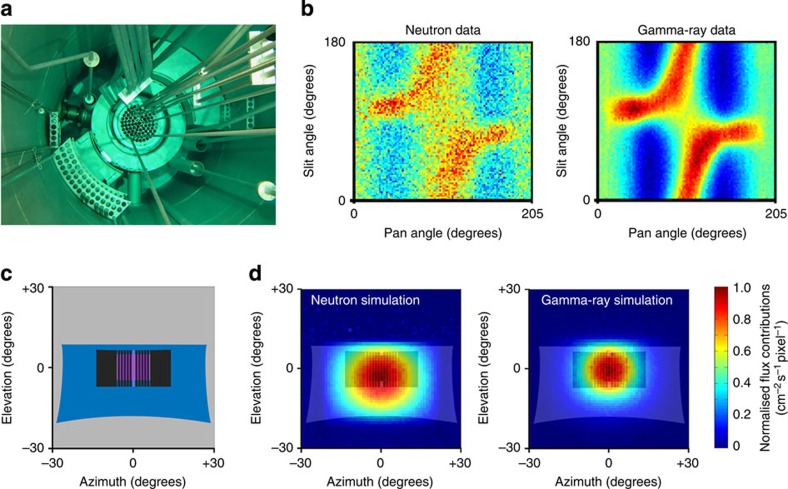
Reactor geometry, data and image simulations. (**a**) Photograph of the TRIGA reactor core taken from the top of the reactor pool. The fuel elements are visible in the centre of the photograph. The probe location would be to the lower left of the photograph. (**b**) Example of experimental data collected by the probe at 40 kW reactor power. The neutron and gamma-ray data were collected simultaneously. (**c**) Colour schematic of the view from the probe at the image origin. This image was used as an underlay to the radiation images as a reference to the surrounding structure including concrete (grey), light water (blue), graphite (black) and enriched-uranium fuel rods (purple). Distortion of these features is due to the preservation of the spherical image output. (**d**) Simulated images of the neutron and gamma-ray fields emitted by the core produced using the pin-hole camera function in MCNP5. The schematic shown in **c** has been used as an underlay for reference to the surrounding structure. These data have been self-normalized in each case.

Images were obtained of the TRIGA mk II training reactor at the Atominstitut, Vienna University of Technology, Austria; a small-scale, pool-type reactor used for training, research and isotope production. The core has an active fuel volume of 70 l and contains 76 fuel elements of uranium–zirconium hydride (UZrH) fuel (plus reflectors). The core is loaded with 3 kg of uranium-235 and has a maximum steady thermal power output of 250 kW corresponding to a peak fast-neutron flux of 4 × 10^12^ cm^−2^ s^−1^ (ref. 21). The core is surrounded by a graphite moderator (which slows neutrons to facilitate the fission process) and a light-water coolant (Figs 1 and 3a).

The imaging method works on the principle of back projection and therefore exploits the tendency of radiation to travel in straight lines in normal circumstances before interaction with the surrounding environment. The rate of detections and the ‘view' of the detector for many orientations are used to determine the origin of the radiation. The detector is surrounded by an interior collimator of tungsten^22^,^23^ and exterior collimator of polyethylene ensuring both radiation types are adequately shielded. The collimator geometry is cylindrical with a 6-mm slit cut into the face of the collimator, resembling a slit pupil, allowing unattenuated passage of radiation through this region (Fig. 2c,d). The resulting unshielded area (the ‘view' of the detector) in the sensitivity map is roughly rectangular in shape, allowing data to be accumulated from a thin band per single measurement (Fig. 2e). This geometry has the advantage of enabling data to be collected from multiple spatial regions simultaneously, reducing data acquisition time whilst maintaining resolution relative to conventional, circular collimator apertures and facilitating use in extreme radiation conditions^24^. As in the eyes of cats, this slit function enables an extension of the useful range to these extreme circumstances.

The reactor was imaged at 40, 100 and 250 kW steady-state reactor powers, ranging from 16 to 100% of the maximum power output. Data were collected in matrix *D* in each case (Fig. 3b). The relationship between the data *D* and the image *I* is expressed as:


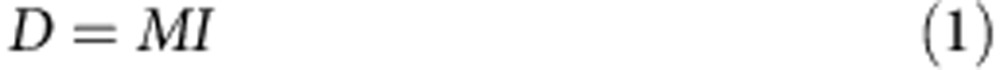


Equation (1) also relates *M*, the sensitivity matrix, a compilation of sensitivity maps for all positions of the slit collimator. The image *I* is then solved iteratively to produce an image with resolution of 1° × 1° bins, which determine the most likely distribution of radiation sources in the surrounding environment given that data *D* was observed. A single iteration is expressed as:


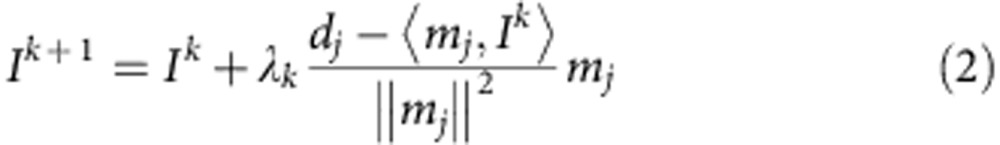


where *d*_*j*_ is the *j*th row of *D*, *m*_*j*_ is the *j*th row of *M* and *λ*_*k*_ is a relaxation parameter. These steps are performed independently for neutrons and gamma rays. It is therefore required that *M* is known for both neutrons and gamma rays for the energy spectra emitted by the reactor. As *M* cannot be calculated easily these matrices were obtained from computer simulations of the slit collimator using the Monte Carlo code MCNP5 (Supplementary Movie 1).

**Figure 4:**
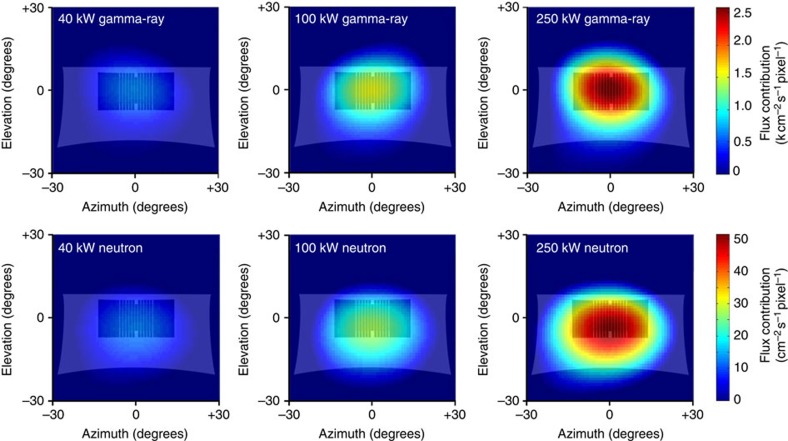
Images of the reactor core. Neutron and gamma-ray images of the reactor core at various steady-state reactor powers overlaid on the schematic shown in Fig. 3c for reference to the surrounding structures. Distortion of the underlay features is due to the preservation of the spherical image output. The bright spots show the origin of the radiation is the fuel rods, and the distributions extend beyond the core in each case implying that scattered radiation contributes to the images. Differences between the neutron and gamma-ray images are present and are attributed to the fundamental differences between neutrons and gamma rays and their interactions within the core structure. In particular it is noticeable that the peak flux of the neutron images appear below the core by a value of 4°; this is due to a combination of two effects. The first is a geometric effect of the image being taken from 25 cm vertically above the core centre, coupled with the presence of the void tunnel. The view through the tunnel is therefore of the core and the region below it. The second effect is the influence of scatter in the graphite moderator. Neutron scattering occurs more frequently, therefore the neutron distribution below the core features more prominently in the image. These images agree closely with Monte Carlo simulations of the set-up.

The solutions of these image data have been overlaid on a sketch of the core from the point of view of the probe (Fig. 4). An alternative normalized scale has also been included for these images (Supplementary Fig. 1). The distortion of the underlay is due to the maintenance of the spherical image, and shows a projection of the fuel elements, graphite shielding and the concrete tunnel (Fig. 3c). The images were compared quantitatively using skewness to aid visual comparison (Supplementary Table 1). These images have been used to produce a video showing what a reactor start-up may look like if imaged frequently over a linear power increase (Supplementary Movie 2).

### Image simulation

A simplified reactor geometry was modelled in MCNP5 comprising a homogeneous core, graphite shielding, light-water moderator, lead shield, cadmium shutter and heavy concrete exterior. To increase computing efficiency, the reactor core was modelled as one homogeneous cylinder containing the appropriate ratios of fuel element and moderating material. The homogeneous core was treated as a volume source producing the appropriate radiation spectrum and distribution. A Watt fission spectrum was used to seed neutrons and the gamma-ray spectrum measured by Verbinksy *et al*.^25^ was used to seed gamma rays. The gamma-ray component due to neutron capture was also included. Each simulation was set-up to closely mimic the data acquisition and the data were presented in the same way using a grid of 1° × 1° bins over the same range (Fig. 3d).

## Discussion

The radiation images of the TRIGA reactor clearly show the location of the reactor core (Fig. 4). The origin of the radiation in each image matches the location of the UZrH fuel rods of the reactor where the fission process is taking place, though it is noticeable that the flux extends beyond the fuel and into the moderator. This result is closely consistent with simulated images of the core (Fig. 3d), and implies that the image depicts the scatter distribution of the radiation in the moderator. Fundamental differences between neutrons and gamma rays can be observed in these images, manifesting in the region of the graphite structure where scattering is most dissimilar. The images were also compared quantitatively by horizontal and vertical weighted skewness (Supplementary Table 1) and were found to be consistent between radiation types and agreed closely with the trends of the simulated results. These images have been plotted on a scale consistent across radiation type and, when viewed together, clearly illustrate the changes in the internal conditions of the reactor. Integrating the flux in each image yields a linear response with reactor power, suggesting that further refinement this technique might be used to quantitatively assess the power distribution and fission rate within the reactor core. An important distinction here is that the gamma-ray image depicts gamma rays from fission and other non-fission reactions alike, whereas the neutron image in this context comprises events arising from ongoing criticalities in fissile material and the interaction of alpha particles on light isotopes in the core (the latter process being a significant contributor to decay heat). The latter would normally be a minority reaction channel and, in any case, would not respond directly to power changes. Contributions from spontaneous fission in ^238^U and even-numbered plutonium isotopes are small. Hence, it is clear that the neutron distribution presented in this research shows the distribution of reacting nuclear fuel exclusively, and the change of this in correspondence with changes in power. Multiple images of this type taken from different locations would allow three-dimensional computer tomography of the neutron and gamma-ray distributions inside the core.

Currently 11% of electricity worldwide is generated by nuclear power; 435 reactors are in operation with 71 new reactors under construction^26^. The majority of these are light-water power reactors or medical reactors which could adopt this technique, giving many opportunities to further our understanding and make reactors safer and more efficient.

## Methods

### Imaging system

The system comprises an imaging probe, mixed-field analyser unit^19^ data acquisition electronics and a PC laptop. The detector is surrounded by an interior tungsten collimator (radius 34 mm), and exterior polyethylene collimator (radius 74 mm). The geometry is cylindrical with a 6-mm wide, 25-mm deep slit-void cut into the face of the tungsten, and a tapered slit in the polyethylene widening from 10 to 20 mm at the outermost radius, also 25 mm deep. The addition of the polyethylene provides additional neutron shielding to improve the neutron sensitivity matrix. This collimator is rotated through two axes by stepper motors located in the probe and controlled by a microcontroller linked to the PC. The axes of rotation are (1) the collimators cylindrical axis (oriented in the horizontal plane) referred to as the slit angle rotation and (2) an orthogonal axis in the vertical referred to as the pan angle rotation. For a single measurement the collimator is rotated to the required position, events from the detector are processed via the mixed-field analyser into neutron and gamma-ray logic pulses. These pulses are then counted over time interval *t*_d_ by binary counters in the data acquisition electronics and transferred to the PC via serial communication where the data is stored.

### Deployment and reactor set-up

The TRIGA reactor is heavily shielded by water and heavy concrete on all sides with exception of an irradiation facility. The primary purpose of the irradiation room is to provide a high flux of neutrons for activation of samples. The energy spectrum of the neutrons can be adjusted by the selective use of a water collimator and a cadmium shield. The water collimator was drained to allow the highest possible flux of fast neutrons and the cadmium shutter was kept in place to limit the activation of the probe from thermal neutrons. The imaging probe and associated equipment was set-up and tested on an elevator serving the irradiation room. The probe was lowered to the level of the reactor core using a service lift, when lowered the detector (that is, the image origin) was located 25 cm vertically above, and 229 cm horizontally from the geometric centre of the reactor core. Power was supplied and data transferred by cabling linking to a PC, mixed-field analyser unit and data acquisition unit set-up at the top of the elevator shaft (Fig. 1).

### Data acquisition and image solution

The image is obtained in two steps: data collection into a data matrix *D* (Fig. 3b), and the solution of the image *I* using an algebraic reconstruction technique^27^. The data matrix comprises many thousands of elements corresponding to different orientations of the slit (and therefore different sensitivity maps), which is rotated mechanically through two axes over approximately a half-universe. The number of radiation events at each orientation is recorded in *D*. Data was collected from 88 slit angles over a 180° rotation, and 108 pan angles corresponding to a 205° rotation centred on the core. Data collection was performed for these 9.5k points with *t*_d_=500 ms at each collimator position that populates D. It should be noted that the image at 250 kW contained 74% of a full data set due to temporary malfunction of the data acquisition software during the imaging routine. However, a full data set is not required for an image solution. The nature of the data collection allowed some information to be collected for every point in the image space, and thus there were no blind spots in the 250 kW image; the only (minor) compromise in this case was on precision, and not accuracy. In fact only a small proportion of these measurements would be needed to form an accurate image solution. The validity in this case is illustrated by the close agreement of all respective neutron and gamma-ray images.

### Determination of system matrix

The data matrix *D* is solved using an algebraic reconstruction technique with a non-negativity constraint. One required component for this solution is the sensitivity matrix *M*, which is more easily obtained from computer simulations rather than experimentation. For this the probe was modelled in the Monte Carlo simulation package MCNP5. A single element of each system matrix was obtained from interrogation of the collimator using fast neutrons and gamma rays of appropriate energy distributions. These simulated results were verified against real experimental results. The radiations were emitted from infinity from all angles around the collimator in 1 × 1° bins allowing the determination of the sensitivity map of the collimated detector to all space. A geometric transformation of these elements was then used to complete the system matrix comprising 16k elements in total, corresponding to all possible orientations of the collimator for the given image parameters (Supplementary Movie 1).

### Image skewness

The images *I* can be compared visually, and can be seen to closely correlate. To allow a metric for quantitative comparison a weighted image skewness was calculated horizontally *S*_H_ and vertically *S*_V_ over the image using the following formulae:


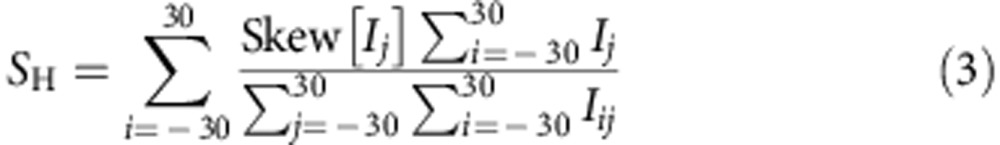



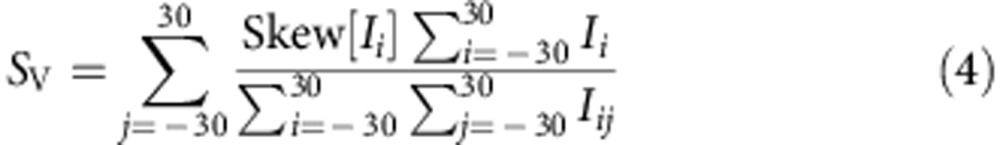


where *i* and *j* refer to the elevation and azimuth angles, respectively. These components of skewness can be assumed to be independent due to the symmetry in the system.

## Additional information

**How to cite this article:** Beaumont, J. S. *et al*. High-intensity power-resolved radiation imaging of an operational nuclear reactor. *Nat. Commun*. 6:8592 doi: 10.1038/ncomms9592 (2015).

## Supplementary Material

Supplementary Information Supplementary Figure 1 and Supplementary Table 1

Supplementary Movie 1 Detector scanning routine expressed through changes in the sensitivity map as the collimator is rotated. The sensitivity maps of the collimated detector to neutrons and gamma-rays in all space are shown, and how they vary as the slit and pan angles change during the scanning routine.

Supplementary Movie 2 Simulated video of a linear power increase in the TRIGA reactor. The radiation images obtained in this work have been used to create additional frames to simulate a video showing a linear power increase in the core from startup to full power. This would be achieved in practise by imaging the core more frequently.
