# Supplementary material for: High-intensity power-resolved radiation imaging of an operational nuclear reactor
Source: Nat Commun. 2015 Oct 9;6:8592. doi: 10.1038/ncomms9592 (PMC4633948; doi:10.1038/ncomms9592)
Supplement: Supplementary Information — Supplementary Figure 1 and Supplementary Table 1 [file ncomms9592-s1.pdf]

Supplementary figures

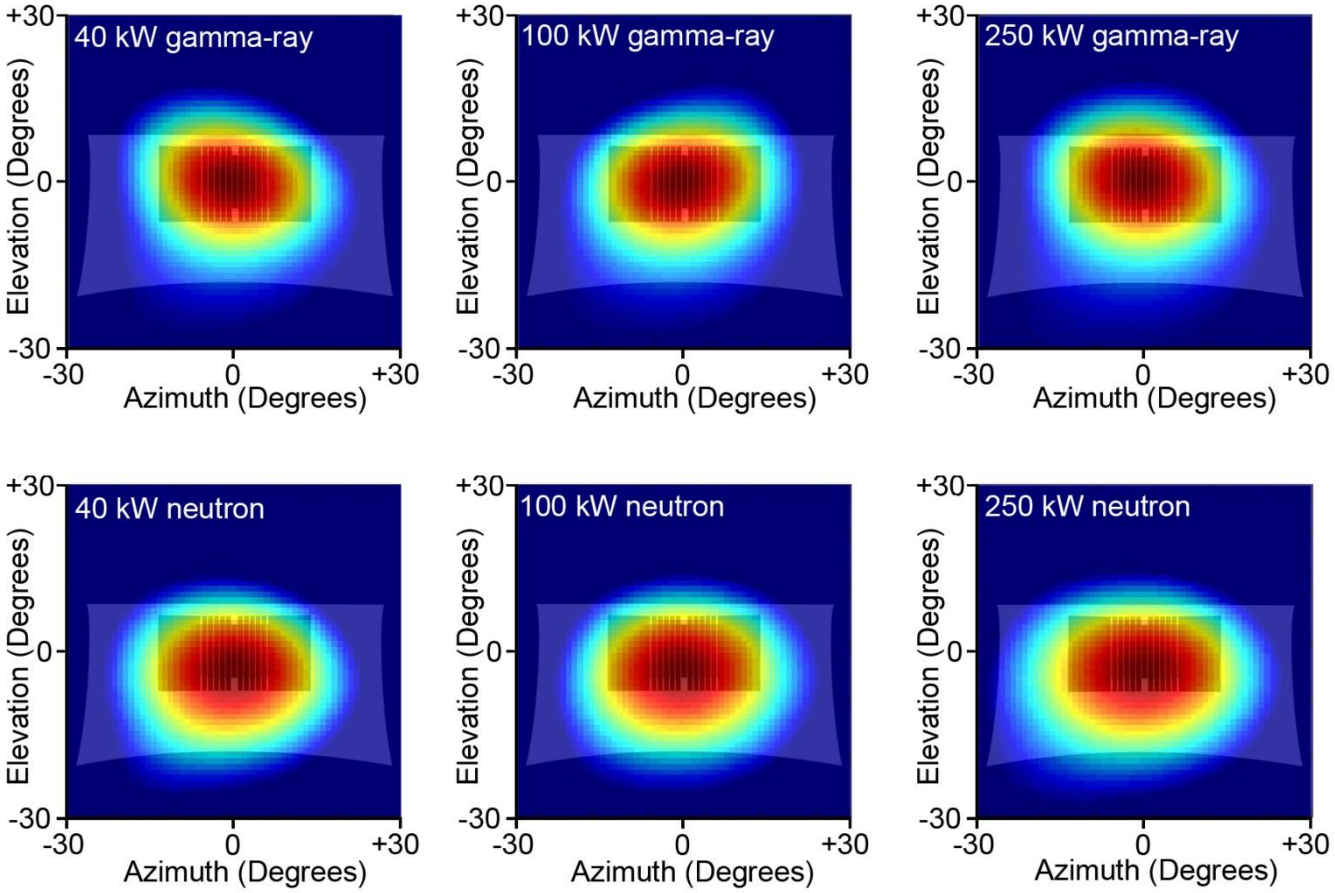

Supplementary Figure 1| Images of the reactor core. Neutron and gamma-ray images of the reactor core at various steady-state reactor powers overlaid on the schematic shown in Fig. 3c for reference to the surrounding structures. Distortion of the underlay features is due to the preservation of the spherical image output. The bright spots show the origin of the radiation is the fuel rods, and the distributions extend beyond the core in each case implying that scattered radiation contributes to the images. Differences between the neutron and gamma-ray images are present and are attributed to the fundamental differences between neutrons and gamma rays and their interactions within the core structure. In particular it is noticeable that the peak flux of the neutron images appear below the core by a value of 4 degrees; this is due to a combination of two effects. The first is a geometric effect of the image being taken from 25 cm vertically above the core centre, coupled with the presence of the void tunnel. The view through the tunnel is therefore of the core and the region below it. The second effect is the influence of scatter in the graphite moderator. Neutron scattering occurs more frequently, therefore the neutron distribution below the core features more prominently in the image. These images agree closely with Monte Carlo simulations of the setup. Here the colour axis has been set by the range in each picture allowing direct comparison.

	Radiation events in data (M)		Peak flux (degrees) [elevation, azimuth]		Flux from core (in image) as fraction of full power value			Weighted horizontal skewness		Weighted vertical skewness	
Image	Neutrons (n)	Gamma (g)	n	g	n	g	Expected	n	g	n	g
40 kW	1.11	44.8	[-4, 0]	[0, 0]	0.207	0.229	0.25	0.118	0.252	0.488	0.565
100 kW	2.49	111	[-4, 0]	[0, 0]	0.470	0.556	0.5	0.071	0.257	0.468	0.549
250 kW	4.76	187	[-4, 0]	[0, 0]	[1]	[1]	1	-0.091	0.172	0.422	0.493
MCNP	-	-	[-4, 0]	[0, 0]	-	-	-	-	-	0.324	0.589

Supplementary Table 1| Table of image parameters. Parameters associated with data collection, solved images and simulated images. Radiation events in data describes the total number of radiation events recorded in a given image, note that the 250 kW value has been adjusted to represent a full data set including data missing due to a temporary electronics malfunction. The peak flux gives the location of the pixel in the image solution with the highest flux. The flux fractions in column four were calculated based on the 250 kW image solution. The weighted skewnesses were calculated according to equation 4.
